# Factors Associated With County-Level Variation in Premature Mortality Due to Noncommunicable Chronic Disease in the United States, 1999-2017

**DOI:** 10.1001/jamanetworkopen.2020.0241

**Published:** 2020-02-28

**Authors:** Suhang Song, Michael G. Trisolini, Kenneth A. LaBresh, Sidney C. Smith, Yinzi Jin, Zhi-Jie Zheng

**Affiliations:** 1 China Center for Health Development Studies, Peking University, Beijing, China; 2 RTI International, Research Triangle Park, North Carolina; 3 Division of Cardiology, School of Medicine, University of North Carolina at Chapel Hill; 4 Department of Global Health, Peking University School of Public Health, Beijing, China; 5 Peking University Institute for Global Health, Beijing, China

## Abstract

**Importance:**

Progress against premature death due to noncommunicable chronic disease (NCD) has stagnated. In the United States, county-level variation in NCD premature mortality has widened, which has impeded progress toward mortality reduction for the World Health Organization (WHO) 25 × 25 target.

**Objectives:**

To estimate variations in county-level NCD premature mortality, to investigate factors associated with mortality, and to present the progress toward achieving the WHO 25 × 25 target by analyzing the trends in mortality.

**Design, Setting, and Participants:**

This cross-sectional study focused on NCD premature mortality and its factors from 3109 counties using US mortality data for cause of death from the Centers for Disease Control and Prevention WONDER databases and county-level characteristics data from multiple databases. Data were collected from January 1, 1999, through December 31, 2017, and analyzed from April 1 through October 28, 2019.

**Exposures:**

County-level factors, including demographic composition, socioeconomic features, health care environment, and population health status.

**Main Outcomes and Measures:**

Variations in county-level, age-adjusted NCD mortality in the US residents aged 25 to 64 years and associations between mortality and the 4 sets of county-level factors.

**Results:**

A total of 6 794 434 deaths due to NCD were recorded during the study period (50.58% women; 16.49% aged 65 years or older). Mortality decreased by 4.30 (95% CI, −4.54 to −4.08) deaths per 100 000 person-years annually from 1999 to 2010 (*P* < .001) and decreased annually at a rate of 0.90 (95% CI, −1.13 to −0.73) deaths per 100 000 person-years annually from 2010 to 2017 (*P* < .001). Mortality in the county with the highest mortality was 10.40 times as high as that in the county with the lowest mortality (615.40 vs 59.20 per 100 000 population) in 2017. Geographic inequality was decomposed by between-state and within-state differences, and within-state differences accounted for most inequality (57.10% in 2017). County-level factors were associated with 71.83% variation in NCD mortality. Association with intercounty mortality was 19.51% for demographic features, 23.34% for socioeconomic composition, 16.40% for health care environment, and 40.75% for health-status characteristics.

**Conclusions and Relevance:**

Given the stagnated trend of decline and increasing variations in NCD premature mortality, these findings suggest that the WHO 25 × 25 target appears to be unattainable, which may be related to broad failure by United Nations members to follow through on commitments of reducing socioeconomic inequalities. The increasing inequalities in mortality are alarming and warrant expanded multisectoral efforts to ameliorate socioeconomic disparities.

## Key Points

**Question:**

How does county-level premature mortality due to noncommunicable chronic disease vary by economic and geographic factors in the United States?

**Findings:**

In this cross-sectional study of 3109 US counties, the variations in mortality due to noncommunicable chronic disease increased from 1999 to 2017, and within-state differences accounted for most of the inequalities (57.10% in 2017). The mortality variation was associated with demographic composition, socioeconomic features, health care environment, and population health status.

**Meaning:**

The increasing variations in premature mortality due to noncommunicable chronic disease suggest a need for expanded efforts across multisectoral actions to reduce the differences in socioeconomic characteristics and prevalence of noncommunicable chronic disease risk factors.

## Teaser

This cross-sectional study examines variation in premature mortality due to noncommunicable chronic disease (NCD) across counties and investigates county-specific factors associated with this mortality among US residents aged 25 to 64 years.

## Introduction

Noncommunicable chronic diseases (NCDs) are the cause in 8 of 10 deaths,^1^ and these rates show substantial and long-standing geographic inequalities in the United States.^2^ More than half of these deaths affect people younger than 70 years.^3^ Premature deaths due to NCD pose large and inequitable health and economic burdens on individuals, societies, and health systems.^4^ The United States agreed to adopt an overarching target of reducing premature mortality from the 4 main NCDs (cardiovascular diseases, chronic respiratory diseases, cancers, and diabetes) by 25% relative to their 2010 levels by 2025 (referred to as the World Health Organization [WHO] 25 × 25 target).^5^ Surveillance studies of NCD mortality in the United States documented temporal trends by race/ethnicity, sex, and age group from 2010 to 2015,^6^ but little is known to date about whether these trends were consistent across counties for different socioeconomic statuses and geographic regions. A national perspective on county-level variation in premature mortality due to the 4 main NCDs could provide insights into regional inequalities and divergent trends in NCD mortality, which could help quantify the magnitude of regional associations with progress toward the WHO 25 × 25 target.

Among persons aged 45 to 79 years, about 50% of NCD mortality is associated with individual-level risk factors such as smoking, hypertension, diabetes, and obesity,^7^ but only about one-tenth of variation in NCD mortality by state is accounted for by these risk factors.^8^ Previous studies reported correlates of NCD mortality, including regional-level or system-related factors such as socioeconomic status, demographic composition, health care access, and environmental features, providing insight into additional characteristics that may predispose certain regions to disadvantage in NCD premature mortality.^9,10,11,12^ A previous study^13^ found that state-level premature cardiac mortality varied considerably across the United States, warranting more detailed studies to identify potential county-specific factors that may be associated with the mortality variation. Despite recognizing the importance of region-specific factors in shaping premature death due to NCDs, quantification of their associations in county-level inequality in death rates is limited by lack of standardized and consistent measures of county-level data linked to county-level NCD premature mortality.

This study aimed to estimate variation in NCD premature mortality across counties in US residents aged 25 to 64 years from January 1, 1999, through December 31, 2017, and to investigate county-specific factors associated with NCD premature mortality. By analyzing the trend in NCD premature mortality, this study also aims to assess the progress toward achieving the WHO 25 × 25 targets.

## Methods

### Data

All data used in this study were deidentified. The Peking University ethics committee did not require institutional review board approval or informed consent for use of these data. This study followed the Strengthening the Reporting of Observational Studies in Epidemiology (STROBE) reporting guideline.

This cross-sectional study defined NCD premature mortality as the number of deaths per 100 000 person-years attributable to cardiovascular diseases, chronic respiratory diseases, cancers, and diabetes that occurred among persons aged 25 to 64 years.^6,14^ County-level, age-adjusted mortality was obtained from the National Center for Health Statistics of the US Centers for Disease Control and Prevention (CDC WONDER databases).^15^ We conducted a cross-sectional study and identified NCD death as having an underlying cause classified in the *International Statistical Classification of Diseases and Related Health Problems, Tenth Revision* (*ICD-10*): cardiovascular disease (*ICD-10* codes I00-I99), chronic respiratory diseases (*IDC-10* codes J40-J47), cancers (*ICD-10* codes C00-C97), and diabetes (*ICD-10* codes E10-E14).^16^

Based on literature review, we included 4 sets of county-specific characteristics that could be associated with NCD mortality variation, including demographic composition, socioeconomic features, health care environment, and population health status. Demographic composition included population size, rurality, sex, age, race/ethnicity, and foreign-born status. Socioeconomic features included median household income, unemployment, school enrollment, and violent crime rate. Health care environment included density of primary care physicians, Medicare enrollees with diabetes undergoing testing of hemoglobin A_1c_ (HbA_1c_) levels, access to places for physical activity, and access to healthy foods. Population health status included NCD risk index, self-rated poor or fair health, and total Medicare reimbursements per enrollee (a measure of use of health care services as a proxy for illness). These data were obtained from multiple databases from 2011 to 2017.^17,18,19,20,21,22,23^ The data sources are outlined in Table 1. Notably, race/ethnicity as a factor influencing the variations in NCD premature mortality was ascertained from death certificates and classified as non-Hispanic white (ie, white), non-Hispanic black or African American (ie, black), and Hispanic or Latino (ie, Latino).

**Table 1.** Data Sources and County Characteristics in the United States

County Characteristic	Data Source	Median (IQR) [Range]
Age-standardized mortality rate from the 4 major NCDs, 1999-2017, deaths per 100 000 population	CDC WONDER^15^	231.10 (185.00 to 287.40) [55.90 to 857.80]
Demographic composition, 2011-2017		
1000 population	CPE^20^	42.41 (22.71 to 106.44) [4.44 to 10 170.29]
Rural, %	UWPHI CHRR^17^^a^	50.56 (26.30 to 71.91) [0 to 100.00]
Female, %	CPE^20^	50.58 (49.87 to 51.21) [34.05 to 56.84]
Aged ≥65 y, %	CPE^20^	16.49 (14.17 to 18.80) [5.10 to 56.94]
African American, %	CPE^20^	3.81 (1.03 to 14.16) [0.05 to 85.33]
Native American/Alaskan, %	CPE^20^	0.51 (0.33 to 1.01) [0.04 to 93.80]
Asian, %	CPE^20^	0.76 (0.46 to 1.58) [0.04 to 43.90]
Hispanic, %	CPE^20^	4.02 (2.10 to 9.06) [0.27 to 96.32]
Born outside the US, %	ACS^21^	2.90 (1.45 to 5.92) [0 to 52.00]
Economic and social features, 2011-2017		
Median household income, $1000	SAIPE^22^	40.77 (34.50 to 48.52) [15.33 to 136.19]
Unemployed, %	BLS^23^	6.39 (4.93 to 8.24) [0.82 to 29.70]
Enrolled in school, %	ACS^21^	24.88 (22.67 to 27.41) [7.86 to 55.16]
No. of violent crimes per 100 000 person-years	UWPHI CHRR^17^^a^	240.65 (145.17 to 390.63) [0 to 2349.64]
Health care and features of the environment		
No. of primary care physicians per 100 000 population, 2011-2017	UWPHI CHRR^17^^a^	52.63 (36.31 to 74.28) [0 to 631.94]
No. of Medicare enrollees aged 65-75 y with diabetes undergoing HbA_1c_ test, 2011-2015^b^	DAHC^18^^c^	85.62 (84.21 to 87.16) [12.28 to 100.00]
People with access to places for physical activity, 2014-2019, %^b^^,^^d^	UWPHI CHRR^17^^a^	61.70 (53.10 to 77.97) [0 to 100.00]
Food environment index, 2014-2019^b^^,^^e^^,^^f^	UWPHI CHRR^17^^a^	7.41 (6.88 to 7.90) [0.50 to 10.00]
Population health indicators		
NCD risk index, 2011-2017^b^^,^^c^^,^^g^	UWPHI CHRR^17^^a^	−0.01 (−1.25 to 1.30) [−7.04 to 5.67]
Poor/fair health, 2011-2017, %	UWPHI CHRR^17^^a^	16.90 (13.50 to 21.00) [3.60 to 50.80]
Total Medicare reimbursements per enrollee, 2011-2016, $1000	DAHC^19^	9.92 (8.89 to 10.38) [4.52 to 17.72]

Abbreviations: ACS, American Community Survey; BLS, Bureau of Labor Statistics; CDC, Centers for Disease Control and Prevention; CHRR, County Health Rankings & Roadmaps; CPE, Census Population Estimates; DAHC, Dartmouth Atlas of Health Care; HbA_1c_, hemoglobin A_1c_; IQR, interquartile range; NCD, noncommunicable chronic diseases; SAIPE, Small Area Income and Poverty Estimates; UWPHI, University of Wisconsin Population Health Institute.

^a^ Provides a model to help communities understand the factors influencing healthy residents and summaries many health outcomes and health factors from other databases each year.

^b^ For those variables restricted to year range, we conducted an ordinary least square regression model to estimate the missing value.

^c^ Indicates a publicly available source of data providing county-level Medicare spending and mortality rates, selected measures of primary care access and quality, and hospital and physician capacity measures.

^d^ The 2014-2019 County Health Rankings and Roadmaps databases were used to summarize this variable from OneSource Global Business Browser (Avention, Concord, Massachusetts), DeLorme map data (DeLorme, Yarmouth, Maine), Esri (Redlands, California), and Census Bureau TIGER/Line files from 2012-2018.

^e^ The 2014-2019 County Health Rankings and Roadmaps databases were used to summarize this variable from US Department of Agriculture Food Environment Atlas, Map the Meal Gap from 2011-2016.

^f^ Calculated as a composite score ranging from 1 to 10, describing limits on access to healthy foods, with 1 indicating the lowest access to healthy foods, and 10 indicating the highest access to healthy foods.

^g^ Calculated by principal components analysis on county-level prevalence of diabetes, tobacco smoking, excessive drinking, obesity, and physical inactivity.

We included all the data available from the CDC WONDER databases. For each year, some counties show unreliable data that cannot be used for analysis because the number of deaths was less than 20. Thus, for 1999 to 2017, the CDC WONDER databases provided reliable data of 3109 counties.

### Statistical Analysis

Data were analyzed from April 1 through October 28, 2019. Given the change in number of counties by year, we estimated the county-level mortality in 2011 through 2017. To examine mortality variation, we estimated the percentage change in county-level mortality from 1999 through 2005 and 2011 through 2017. A total of 3024 counties were included in 1999 through 2005 and 3027 in 2011 through 2017. To decompose the geographic variations, we conducted the Theil index of county-level mortality. The advantage of the Theil index is that it can decompose the inequality into within- and between-state inequality.^24,25,26^ We also assessed differences in the trends of mortality by income groups, 4 US regions (Midwest, Northeast, South, and West), and race/ethnicity from 1999 to 2017. The mortality trends were tested using interrupted time series to identify whether mortality varied significantly by year. Mortality rates in our study were age adjusted to be standardized to the 2000 US population.

We used hierarchical linear mixed models to estimate the associations of county-specific factors with NCD premature mortality. To quantify the extent to which the 4 sets of county-specific characteristics were associated with mortality variation, we conducted the dominance analysis for decomposition by examining the relative importance of these variables in contributing to the *R*^2^ value of the regression. We also fitted 4 models by sequentially adding sets of characteristics, based on the notion that we structured the analysis to account first for nonmodifiable factors and subsequently for modifiable factors. Coefficients in the final model can be interpreted as county-level mortality associated with each domain after included nonmodifiable and modifiable factors have been accounted for. For sensitivity analysis, we calculated the annual percentage rate for the trend analysis except for interrupted time series. We conducted an ordinary least square regression to explore the association between mortality and other factors except for hierarchical linear mixed models, and the results were robust. All analyses were conducted with STATA MP, version 14.0 (StataCorp LLC). Two-sided *P* < .05 indicated significance.

## Results

### Trends in NCD Premature Mortality

A total of 3109 counties had 6 794 434 premature deaths due to NCD recorded from January 1, 1999, through December 31, 2017. Among these, 50.58% (interquartile range [IQR], 49.87%-51.21%) were women, 49.42% (IQR, 48.79%-50.13%) were men, 16.49% (IQR, 14.17%-18.80%) were 65 years or older, and 50.56% (IQR, 26.30%-71.91%) lived in rural areas. Table 2 summarizes the results for the 4 NCDs from 1999 to 2017 at the national level. Mortality decreased annually by 4.30 (95% CI, −4.54 to −4.08) deaths per 100 000 person-years from 1999 to 2010 (*P* < .001) and decreased annually at a rate of 0.90 (95% CI, −1.13 to −0.73) deaths per 100 000 person-years from 2010 to 2017 (*P* < .001). The age-adjusted NCD mortality decreased by 22.10% from 1999 to 2017, and mortality due to cardiovascular diseases and cancers decreased by 21.70% and 27.10%, respectively. Mortality due to chronic respiratory diseases slightly decreased by 0.04% and mortality due to diabetes slightly increased by 0.04% (Table 2).

**Table 2.** Trends and Variation in Age-Adjusted Mortality Rate Due to NCDs in US Residents Aged 25-64 Years, 1999-2017

Study Year	Mortality Rate by NCD, Deaths per 100 000 Population (SE)					County-Level Variation in Mortality Rate, Theil Index		
	All^a^	Cardiovascular Diseases	Chronic Respiratory Diseases	Cancers	Diabetes	Overall	Within-State Contribution (%)	Between-State Contribution (%)
1999	232.70 (0.40)	100.20 (0.30)	11.00 (0.10)	110.10 (0.30)	11.50 (0.10)	0.037	0.023 (62.20)	0.014 (37.80)
2000	229.00 (0.40)	98.50 (0.30)	10.50 (0.10)	108.50 (0.30)	11.50 (0.10)	0.039	0.025 (64.10)	0.014 (35.90)
2001	224.50 (0.40)	95.40 (0.30)	10.50 (0.10)	107.00 (0.30)	11.60 (0.10)	0.040	0.025 (62.50)	0.015 (37.50)
2002	221.10 (0.40)	94.50 (0.20)	10.30 (0.10)	104.60 (0.30)	11.80 (0.10)	0.041	0.024 (58.50)	0.017 (41.50)
2003	217.30 (0.40)	92.70 (0.20)	10.40 (0.10)	102.40 (0.30)	11.90 (0.10)	0.042	0.025 (59.50)	0.017 (40.50)
2004	209.00 (0.40)	88.60 (0.20)	9.70 (0.10)	99.20 (0.20)	11.50 (0.10)	0.048	0.028 (58.30)	0.020 (41.70)
2005	207.10 (0.30)	87.50 (0.20)	10.20 (0.10)	97.90 (0.20)	11.50 (0.10)	0.048	0.028 (58.30)	0.020 (41.70)
2006	202.20 (0.30)	85.30 (0.20)	9.60 (0.10)	95.90 (0.20)	11.30 (0.10)	0.047	0.028 (59.60)	0.019 (40.40)
2007	197.00 (0.30)	82.60 (0.20)	9.70 (0.10)	93.80 (0.20)	10.90 (0.10)	0.047	0.028 (59.60)	0.019 (40.40)
2008	194.40 (0.30)	81.40 (0.20)	10.20 (0.10)	92.20 (0.20)	10.60 (0.10)	0.048	0.027 (56.20)	0.021 (43.70)
2009	191.70 (0.30)	79.50 (0.20)	10.10 (0.10)	91.60 (0.20)	10.50 (0.10)	0.051	0.029 (56.90)	0.022 (43.10)
2010	188.90 (0.30)	78.20 (0.20)	9.80 (0.10)	90.50 (0.20)	10.40 (0.10)	0.051	0.029 (56.90)	0.022 (43.10)
2011	187.50 (0.30)	77.40 (0.20)	10.00 (0.10)	89.10 (0.20)	10.90 (0.10)	0.053	0.031 (58.50)	0.022 (41.50)
2012	186.10 (0.30)	77.10 (0.20)	10.00 (0.10)	88.30 (0.20)	10.60 (0.10)	0.056	0.032 (57.10)	0.024 (42.90)
2013	185.30 (0.30)	77.30 (0.20)	10.30 (0.10)	86.70 (0.20)	11.00 (0.10)	0.055	0.031 (56.40)	0.024 (43.60)
2014	185.00 (0.30)	77.70 (0.20)	10.30 (0.10)	85.80 (0.20)	11.10 (0.10)	0.059	0.033 (55.90)	0.026 (44.10)
2015	184.20 (0.30)	78.10 (0.20)	10.50 (0.10)	84.10 (0.20)	11.60 (0.10)	0.061	0.035 (57.40)	0.026 (42.60)
2016	183.70 (0.30)	78.90 (0.20)	10.60 (0.10)	82.70 (0.20)	11.60 (0.10)	0.061	0.036 (59.00)	0.025 (41.00)
2017	181.30 (0.30)	78.50 (0.20)	10.60 (0.10)	80.30 (0.20)	12.00 (0.10)	0.063	0.036 (57.10)	0.027 (42.90)

Abbreviation: NCD, noncommunicable chronic disease.

^a^ The trend of mortality was tested by interrupted time series. The mortality decreased annually by 4.30 (95% CI, −4.54 to −4.08; *P* < .001) deaths per 100 000 person-years from 1999 to 2010 and decreased annually at a rate of 0.90 (95% CI, −1.13 to −0.73, *P* < .001) deaths per 100 000 person-years from 2010 to 2017.

### Trends in Variations in NCD Premature Mortality

**Figure 1.**
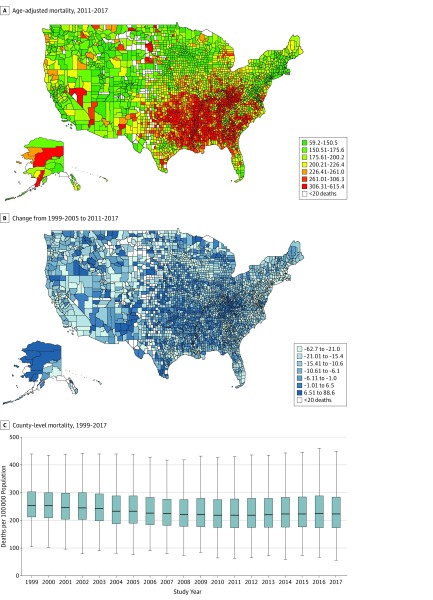
County-Level Variation in Age-Adjusted Premature Mortality Due to Noncommunicable Chronic Diseases Data are obtained from US counties from January 1, 1999, through December 31, 2017, for residents aged 25 to 64 years. A, Age-adjusted mortality from the 4 major noncommunicable chronic diseases in 3147 counties, 2011 through 2017. Data are shown as mortality per 100 000 person-years. B, Data are shown as percentage of change in the age-adjusted mortality by 3024 counties from 1999 through 2005 and 3027 counties from 2011 through 2017. C, Data are adjusted for age. Boxes indicate interquartile range; error bars, range; and horizontal lines, median. Counties with less than 20 deaths were not included in the analysis.

The county with the highest mortality had a rate 10.40 times as high as that of the county with the lowest mortality (615.40 vs 59.20 per 100 000 population) in 2017. The highest rates in 2011 to 2017 were observed in counties in a band, stretching from Oklahoma to Mississippi and eastern Kentucky. Conversely, the lowest rates were observed in counties in central Colorado and near the border of Idaho, Montana, and Wyoming (Figure 1A). The percentage change in mortality between 1999 to 2005 and 2011 to 2017 varied across counties. In particular, increasing rates of mortality were observed in many of the same counties in the band of South-Central states that had the highest mortality in 2011 to 2017 (Figure 1B). The county-level mortality from 1999 to 2017 (Figure 1C) showed similar trends to the national-level mortality (Table 2). There was a deflection point in both trends in 2010, with significant slowing in the decline from 2010 to 2017.

For the county-level variation in mortality, the Theil index increased from 0.037 in 1999 to 0.063 in 2017, indicating an increase in county-level mortality inequality. The geographic inequalities were decomposed between states and within states, and within-state differences accounted for most of the inequalities (57.10% in 2017). The degree of between-state inequality increased faster than the rate of within-state inequality, and the proportion of overall within-state inequality thus declined by year (Table 2).

**Figure 2.**
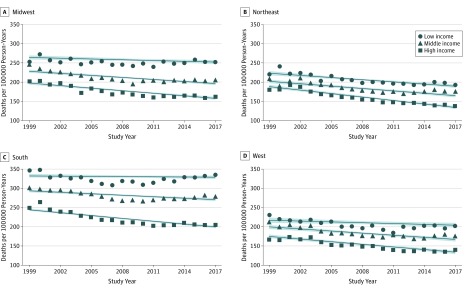
Trends in County-Level, Age-Adjusted Premature Mortality Due to Noncommunicable Chronic Diseases by Income Groups Includes US residents aged 25 to 64 years by income groups in 4 US regions (Midwest, Northeast, South, and West) from January 1, 1999, through December 31, 2017. Fitted line indicates median; shaded area, 95% CI.

Premature mortality due to NCD also varied by regions and income groups, as shown in Figure 2. The mortality rates among low-income counties were significantly higher than those among high-income counties, and the gaps between low- and high-income counties were widened in all 4 US regions. The median mortality for low-income counties in 2017 was 301.90 per 100 000 person-years; for middle-income counties, 224.30 per 100 000 person-years; and for high-income counties, 164.90 per 100 000 person-years. In comparison, these rates were 315.55 for low-income counties, 252.80 for middle-income counties, and 212.70 for high-income counties in 1999. During 2010 and 2017, high-income counties showed declines in trends of mortality (1.42% in the Midwest, 6.31% in the Northeast, 1.49% in the South, and 2.38% in the West), while low-income counties had increased rates of mortality in 3 regions (1.16% in the Midwest, 8.33% in the South, and 5.26% in the West) and a 3.01% decrease in the Northeast (Figure 2). Mortality variation by race/ethnicity is shown in eFigure in the Supplement. The trends in mortality were tested by interrupted time series in eTables 1 and 2 in the Supplement.

### Associations Between County Characteristics and NCD Premature Mortality

For bivariate associations, county-level mortality was independently associated with demographic composition, socioeconomic features, health care environment, and population health status. The 4 sets of factors collectively were associated with 71.83% mortality variation (model 4). Dominance analysis showed that demographic composition had a 19.51% association with the mortality variation; socioeconomic features, 23.34%; health care environment, 16.40%; and population health status, 40.75% (Table 3).

**Table 3.** County-Level Factors Associated With Age-Adjusted NCD Death Rates in US Residents Aged 25-64 Years From 2011 to 2017

County Characteristic	Standard Dominance Statistic, %^a^	Coefficient (95% CI)^a^				
		Bivariate Associations	Model 1	Model 2	Model 3	Model 4
Demographic composition						
1000 population	19.51	−0.04 (−0.04 to −0.04)	0.004 (0.001 to 0.01)	−0.002 (−0.004 to 0.001)	−0.001 (−0.004 to 0.001)	−0.003 (−0.005 to 0.001)
Rural, %		1.09 (1.04 to 1.14)	0.51 (0.46 to 0.56)	0.23 (0.18 to 0.28)	0.15 (0.10 to 0.21)	0.06 (0.01 to 0.11)
Female, %		1.45 (0.65 to 2.26)	−1.33 (−1.94 to −0.71)	0.19 (−0.38 to 0.76)	0.55 (−0.03 to 1.12)	−0.45 (−0.99 to 0.09)
Age ≥65 y, %		3.03 (2.70 to 3.35)	2.62 (2.33 to 2.91)	−0.96 (−1.31 to −0.60)	−0.74 (−1.10 to −0.37)	0.25 (−0.10 to 0.59)
African American, %		2.18 (2.09 to 2.27)	2.12 (2.03 to 2.22)	1.12 (1.03 to 1.22)	0.95 (0.85 to 1.06)	0.63 (0.53 to 0.73)
Native American/Alaskan, %		1.27 (1.01 to 1.53)	1.50 (1.29 to 1.71)	0.79 (0.60 to 0.98)	0.57 (0.36 to 0.77)	0.11 (−0.09 to 0.31)
Asian, %		−8.14 (−8.54 to −7.73)	−0.003 (−0.60 to 0.59)	3.16 (2.63 to 3.70)	3.23 (2.70 to 3.76)	2.17 (1.69 to 2.65)
Hispanic, %		−1.38 (−1.49 to −1.27)	1.27 (1.08 to 1.46)	0.32 (0.15 to 0.49)	0.25 (0.08 to 0.42)	−0.34 (−0.50 to −0.18)
Born outside the US, %		−5.02 (−5.24 to −4.81)	−3.87 (−4.32 to −3.43)	−2.68 (−3.07 to −2.28)	−2.45 (−2.85 to −2.06)	−1.61 (−1.98 to −1.24)
Economic and social features						
Median household income, $1000	23.34	−3.81 (−3.88 to −3.73)	NA	−2.53 (−2.64 to −2.43)	−2.42 (−2.52 to −2.31)	−1.52 (−1.63 to −1.40)
Unemployed, %		9.00 (8.46 to 9.54)	NA	−0.32 (−0.76 to 0.11)	−0.82 (−1.27 to −0.37)	−0.91 (−1.33 to −0.48)
Enrolled in school, %		−4.28 (−4.59 to −3.97)	NA	−3.33 (−3.65 to −3.02)	−3.28 (−3.6 to −2.96)	−1.55 (−1.86 to −1.24)
No. of violent crimes per 100 000 population		0.09 (0.08 to 0.09)	NA	0.02 (0.02 to 0.03)	0.02 (0.02 to 0.03)	0.02 (0.02 to 0.03)
Health care and features of the environment						
No. of primary care physicians per 100 000 population	16.40	−0.71 (−0.75 to −0.67)	NA	NA	−0.12 (−0.15 to −0.09)	0.005 (−0.02 to 0.03)
No. of Medicare enrollees aged 65-75 y with diabetes undergoing HbA_1c_ test, %		−3.21 (−3.51 to −2.92)	NA	NA	−0.31 (−0.54 to −0.09)	−0.11 (−0.33 to 0.10)
Access to exercise opportunities, %		−1.76 (−1.82 to −1.69)	NA	NA	−0.12 (−0.17 to −0.06)	−0.03 (−0.09 to 0.02)
Food environment index		−39.47 (−40.72 to −38.22)	NA	NA	−4.02 (−5.48 to −2.56)	−1.23 (−2.60 to 0.14)
Population health indicators						
NCD risk index	40.75	34.40 (33.90 to 34.90)	NA	NA	NA	12.3 (11.14 to 13.47)
Poor or fair health, %		10.15 (9.95 to 10.35)	NA	NA	NA	2.23 (1.95 to 2.52)
Total Medicare reimbursements per enrollee, $1000		26.26 (25.26 to 27.27)	NA	NA	NA	7.63 (6.77 to 8.49)
Variance components						
State intercept variance	NA	NA	1080.54 (NA)	794.55 (NA)	750.74 (NA)	193.37 (NA)
Model residual variance	NA	NA	2557.71 (NA)	1964.48 (NA)	1944.38 (NA)	1702.87 (NA)
Variance modeled	NA	NA	57.68 (NA)	67.50 (NA)	67.83 (NA)	71.83 (NA)
						
Model fit, log likelihood	NA	NA	−64 943.40 (NA)	−63 341.10 (NA)	−63 277.50 (NA)	−62 443.10 (NA)
Adjusted *R*^2^ value	NA	NA	0.40 (NA)	0.57 (NA)	0.59 (NA)	0.69 (NA)

Abbreviations: HbA_1c_, hemoglobin A_1c_; NA, not applicable; NCD, noncommunicable chronic disease.

^a^ We combined the data on mortality from the Centers for Disease Control and Prevention WONDER databases and data on county characteristics from the multiple county-level databases in 2011 to 2017. For hierarchical linear mixed models, 87 counties were excluded owing to lack of data from the multiple county-level databases. Four variables (Medicare enrollees with diabetes undergoing HbA_1c_ testing, access to places for physical activity, food environment index, and total Medicare reimbursements per enrollee) restricted to year range. For those variables, we conducted an ordinary least square regression model to estimate the missing value.

In model 1, for each 1-point increase in the percentage of African American residents, mortality was higher by 2.12 (95% CI, 2.03-2.22) deaths per 100 000 person-years; for each 1-point increase in the percentage of Native American/Alaskan residents, mortality was higher by 1.50 (95% CI, 1.29-1.71) deaths per 100 000 person-years. For each 1-point increase in the percentage of foreign-born residents, mortality was lower by 3.87 (95% CI, −4.32 to −3.43) deaths per 100 000 person-years. In model 4, mortality association for median household income (thousands of dollars) was −1.52 (95% CI, −1.63 to −1.40) deaths per 100 000 person-years; for percentage of unemployment, −0.91 (95% CI, −1.33 to 0.48) deaths per 100 000 person-years; for percentage of school enrollment, −1.55 (95% CI, −1.86 to −1.24) deaths per 100 000 person-years; and for violent crime rate, 0.02 (95% CI, 0.02-0.03) deaths per 100 000 person-years. For the health care environment factor, the percentage of Medicare enrollees with diabetes who underwent HbA_1c_ testing was inversely associated with mortality at −0.11 (95% CI, −0.33 to 0.10) deaths per 100 000 person-years, and the food environment index was negatively associated with mortality at −1.23 (95% CI, −2.60 to 0.14) deaths per 100 000 person-years. For the population health status factor, the NCD risk index was positively associated with mortality at 12.3 (95% CI, 11.14-13.47) deaths per 100 000 person-years; percentage of population with poor or fair health, 2.23 (95% CI, 1.95-2.52) deaths per 100 000 person-years; and total Medicare reimbursements per enrollee, 7.63 (95% CI, 6.77-8.49) deaths per 100 000 person-years.

## Discussion

In this cross-sectional study of 3109 US counties, the variation in NCD premature mortality increased from 1999 to 2017, with in-state differences accounting for most of the inequalities. Mortality variation was associated with demographic composition, socioeconomic features, health care environment, and population health status. The slowing trend in the decrease of mortality and widening variations in mortality suggest the importance of the analysis on the influencing factors for mortality disadvantage.

The findings of the present study indicated that NCD premature deaths were heavily clustered in counties with low socioeconomic status (eg, low income and high proportion of black people).^27^ In addition, the South had higher NCD mortality rates than other regions. Geographic inequalities in mortality were associated with factors related to measures of socioeconomic context (eg, high rate of violent crime).^28^ Many southern counties are impoverished and medically underserved and experience other negative health outcomes.^29^ The South, with high prevalence of smoking, diabetes, and obesity, has lagged behind the rest of the United States in improving these measures.^30,31^ Moreover, within-state differences accounted for most of the geographic inequalities. By way of explanation, factors related to socioeconomic context may have more diversity within states than across states. However, the proportion of within-state inequality declined by year, which called our attention to policy changes focusing on efforts to reduce within-state and across-state socioeconomic inequality. Therefore, it appears that future policies, programs, and nationwide campaigns should consider more focused efforts in countries with a lower socioeconomic status, potentially targeting factors that may be associated with the differences in outcomes within states.

The near doubling of the magnitude of geographic inequality in NCD premature mortality was concerning. Nevertheless, we further found significant declines of mortality among high-income counties and significant increases in low-income counties, widening the gap between the high- and low-income counties from 2010 to 2017. The divergent trends have several explanations. High-income counties may have a greater capacity to quickly adopt new models of health care delivery, join campaigns to reduce NCD, and implement evidence-based recommended actions for primary and secondary treatment.^32^ In addition, high-income counties may have greater resources to invest in the physical and social health environment.^33^ Conversely, low-income counties may face unique challenges (eg, exposure to stress, inadequate social support), disorganized health services, or poor responsiveness of health systems that could attenuate the success of new prevention efforts to reduce NCD.^34^ These challenges are consistent with previous studies that demonstrated that sociodemographic and geographic gaps may widen when large declines in diseases occur with successful established prevention activities, as is true for NCDs.^35^ Theses studies argue that the ability to benefit from advances in disease prevention policies and programs is unequally distributed according to the socioeconomic standing of individuals and communities, thereby leading to differential rates of decline and subsequent widening of inequalities. Thus, the strategies for NCD control and prevention (ie, interventions aimed at community-level changes in health care access) should have been noted, given the differential resources available to communities and counties to make such interventions effective and sustain them.

Because socioeconomic status was associated with the widening mortality variations, we quantified population health status and socioeconomic features that might largely explain mortality variation, whereas health care environment added little information. These results provide insight into which characteristics may predispose certain counties to NCD mortality disadvantage and further imply that reducing inequalities across counties might likely require policies aimed at improving the socioeconomic circumstances of disadvantaged counties. Moreover, access to health care may not influence mortality inequalities as much as poor health status in the first place, making primordial prevention of risk factors a primary health-related goal for reducing geographic inequalities in NCD mortality.^36^ Because the mortality inequalities are largely associated with socioeconomic factors beyond the scope of traditional public health, we may be placing an unrealistic burden on health departments to reduce the mortality gap between counties.

Although mortality inequalities are largely associated with socioeconomic factors, we found that NCD mortality in the South showed similar trends across counties of different income levels, suggesting that the influence of high-income status on reducing the NCD burden had not reached the disadvantaged geographic pattern. For example, people living in high-income counties tend to have higher Medicare reimbursements but less access to NCD treatment and prevention programs if they reside in the South, which leads to higher risk of NCD death. Moreover, among 19 states that did not join Medicaid expansion, 10 of them were in the South.^37^ Lack of Medicaid expansion may obstruct access to health care, especially for people with low socioeconomic status in the South. A prior study showed that income difference may be associated with delayed and forgone health care.^38^ Thus, lack of Medicaid expansion may be a potential factor underlying income-based inequalities. In addition, southern counties have higher proportions of black people, which may relate to other county-level factors (eg, health behaviors, health care access, and physical environment).^39^ Thus, it appears that improving income status would not be enough to reduce the NCD burden in a racially disadvantaged population.

Our findings suggest that health policies aimed at reducing variations in NCD premature mortality require an understanding of modifiable socioeconomic status. That understanding is critical for identifying specific interventions and policies (eg, Medicaid expansion in the South, community-based health care for the black population) to ameliorate socioeconomic inequalities with the goal of addressing mortality inequalities and is an important area for future research. The implications of this study may provide lessons for other countries making progress toward achieving the WHO 25 × 25 target, which may help inspire joint actions on a large scale.

### Limitations

This study had several limitations. First, the observational nature of our study limited our ability to draw any causal inference from our findings. Second, there was missingness of counties for each year. Some counties had unreliable data that cannot be used for analysis because the number of deaths was less than 20. To avoid bias, we estimated the mortality in 2011 to 2017 for 3027 of 3147 counties. To examine the trend in mortality variation, we estimated the percentage change in county-level mortality from 1999 to 2005 and from 2011 to 2017. Third, county-specific factors could only be obtained from 2011 to 2017, and the access to data was even worse for some variables. However, for those variables, we conducted an ordinary least square regression analysis to estimate the missing value. Moreover, owing to the limitation of the number of county-specific factors, the percentages of associations between premature mortality and each domain were potentially biased. To reduce bias, we conducted a factor analysis to explore the factors associated with mortality and finally included as many as 20 influencing factors from multiple databases that exist in every domain.

## Conclusions

Given the stagnated trend of decline and increasing variations in NCD premature mortality, this study suggest that the WHO 25 × 25 target appears to be unattainable, which may be related to broad failure by United Nations members to follow through on commitments of reducing the socioeconomic inequalities. The increasing inequalities in mortality are alarming and warrant expanded multisectoral efforts to ameliorate socioeconomic disparities.

## Notes

Supplement. **eFigure.** Trends in County-Level, Age-Adjusted NCD Premature Mortality by Race/Ethnicity Groups, 1999-2017 **eTable 1.** Results of ITS on Trends in County-Level, Age-Adjusted NCD Premature Mortality by Income Groups in 4 US Regions, 1999-2017 **eTable 2.** Results of ITS on Trends in County-Level, Age-Adjusted NCD Premature Mortality by Race/Ethnic Groups, 1999-2017

## References

[1] Bauer UE, Briss PA, Goodman RA, Bowman BA Prevention of chronic disease in the 21st century: elimination of the leading preventable causes of premature death and disability in the USA. Lancet. 2014;384(9937):-. doi:10.1016/S0140-6736(14)60648-6 24996589

[2] Dwyer-Lindgren L, Bertozzi-Villa A, Stubbs RW, US county-level trends in mortality rates for major causes of death, 1980-2014. JAMA. 2016;316(22):2385-2401. doi:10.1001/jama.2016.13645 27959996 PMC5576343

[3] World Health Organization Global Health Estimates 2015: Deaths by Cause, Age, Sex, by Country and by Region, 2000-2015. Geneva, Switzerland: World Health Organization; 2016.

[4] Di Cesare M, Khang YH, Asaria P, ; Lancet NCD Action Group Inequalities in non-communicable diseases and effective responses. Lancet. 2013;381(9866):585-597. doi:10.1016/S0140-6736(12)61851-0 23410608

[5] Bennett JE, Stevens GA, Mathers CD, ; NCD Countdown 2030 collaborators NCD Countdown 2030: worldwide trends in non-communicable disease mortality and progress towards Sustainable Development Goal target 3.4. Lancet. 2018;392(10152):1072-1088. doi:10.1016/S0140-6736(18)31992-5 30264707

[6] Shiels MS, Berrington de González A, Best AF, Premature mortality from all causes and drug poisonings in the USA according to socioeconomic status and rurality: an analysis of death certificate data by county from 2000-15. Lancet Public Health. 2019;4(2):e97-e106. doi:10.1016/S2468-2667(18)30208-1 30655229 PMC6392082

[7] Patel SA, Winkel M, Ali MK, Narayan KM, Mehta NK Cardiovascular mortality associated with 5 leading risk factors: national and state preventable fractions estimated from survey data. Ann Intern Med. 2015;163(4):245-253. doi:10.7326/M14-1753 26121190

[8] Patel SA, Narayan KMV, Ali MK, Mehta NK Interstate variation in modifiable risk factors and cardiovascular mortality in the United States. PLoS One. 2014;9(7):e101531. doi:10.1371/journal.pone.0101531 25003975 PMC4086813

[9] Patel SA, Ali MK, Narayan KMV, Mehta NK County-level variation in cardiovascular disease mortality in the United States in 2009-2013: comparative assessment of contributing factors. Am J Epidemiol. 2016;184(12):933-942. doi:10.1093/aje/kww081 27864183

[10] Dwyer-Lindgren L, Bertozzi-Villa A, Stubbs RW, Trends and patterns of differences in chronic respiratory disease mortality among US counties, 1980-2014. JAMA. 2017;318(12):1136-1149. doi:10.1001/jama.2017.11747 28973621 PMC5818814

[11] Withrow DR, de González AB, Spillane S, Trends in mortality due to cancer in the US by age and county-level income, 1999-2015 [published online June 14, 2019]. J Natl Cancer Inst. doi:10.1093/jnci/djz123 31199459 PMC6695317

[12] Shrestha SS, Thompson TJ, Kirtland KA, Changes in disparity in county-level diagnosed diabetes prevalence and incidence in the United States, between 2004 and 2012. PLoS One. 2016;11(8):e0159876. doi:10.1371/journal.pone.0159876 27487006 PMC4972249

[13] Alwan A, Maclean DR, Riley LM, Monitoring and surveillance of chronic non-communicable diseases: progress and capacity in high-burden countries. Lancet. 2010;376(9755):1861-1868. doi:10.1016/S0140-6736(10)61853-3 21074258

[14] Chen Y, Freedman ND, Albert PS, Association of cardiovascular disease with premature mortality in the United States. JAMA Cardiol. 2019;4(12):1230-1238. doi:10.1001/jamacardio.2019.3891 31617863 PMC6802055

[15] National Center for Health statistics, Centers for Disease Control and Prevention. About underlying cause of death, 1999-2017. CDC WONDER. Atlanta, GA: Centers for Disease Control and Prevention; 2019. https://wonder.cdc.gov/ucd-icd10.html. Accessed June 20, 2019.

[16] World Health Organization Fact sheets on sustainable development goals: noncommunicable diseases 2017 http://www.euro.who.int/en/health-topics/health-policy/sustainable-development-goals/publications/2017/fact-sheets-on-sustainable-development-goals-health-targets/fact-sheet-on-sdgs-noncommunicable-diseases-sdg-target-3.4. Published 2017. Accessed June 20, 2019.

[17] University of Wisconsin Population Health Institute County Health Rankings & Roadmaps 2010-2019. https://uwphi.pophealth.wisc.edu/chrr/. Published 2019. Accessed June 20, 2019.

[18] Dartmouth Atlas of Health Care Selected measures of primary care access and quality (county level). https://www.dartmouthatlas.org/downloads/tables/PC_County_rates_2015.xls. Published 2019. Accessed June 20, 2019.

[19] Dartmouth Atlas of Health Care Medicare spending: price, age, sex and race-adjusted claims-based data (county level). https://www.dartmouthatlas.org/downloads/tables/pa_reimb_county_2016.xls. Published 2019. Accessed June 20, 2019.

[20] US Census Bureau Census Population Estimates. https://factfinder.census.gov/faces/nav/jsf/pages/searchresults.xhtml?refresh=t. Published 2019. Accessed June 20, 2019.

[21] US Census Bureau American Community Survey, 5-year estimates. American FactFinder. https://factfinder.census.gov/faces/nav/jsf/pages/download_center.xhtml. Published 2019. Accessed December 12, 2019.

[22] US Census Bureau Small area income and poverty estimates. https://www.census.gov/programs-surveys/saipe/data/datasets.html. Published 2019. Accessed June 20, 2019.

[23] Bureau of Labor Statistics County data: labor force data by county. https://www.bls.gov/lau/#cntyaa. Published 2019. Accessed June 20, 2019.

[24] Anand S, Fan VY, Zhang J, China’s human resources for health: quantity, quality, and distribution. Lancet. 2008;372(9651):1774-1781. doi:10.1016/S0140-6736(08)61363-X 18930528

[25] Song S, Hou Z, Zhang L, Meng Q, Kawachi I Can equitable distribution of health resources reduce under-five mortality rate? a cross-sectional study with multilevel analysis of rural counties in China. Lancet. 2018;392(suppl 1):S58. doi:10.1016/S0140-6736(18)32687-4

[26] Rossen LM, Schoendorf KC Trends in racial and ethnic disparities in infant mortality rates in the United States, 1989-2006. Am J Public Health. 2014;104(8):1549-1556. doi:10.2105/AJPH.2013.301272 24028239 PMC4103228

[27] Niessen LW, Mohan D, Akuoku JK, Tackling socioeconomic inequalities and non-communicable diseases in low-income and middle-income countries under the Sustainable Development agenda. Lancet. 2018;391(10134):2036-2046. doi:10.1016/S0140-6736(18)30482-3 29627160

[28] Sommer I, Griebler U, Mahlknecht P, Socioeconomic inequalities in non-communicable diseases and their risk factors: an overview of systematic reviews. BMC Public Health. 2015;15(1):914. doi:10.1186/s12889-015-2227-y 26385563 PMC4575459

[29] Kaplan GA Socioeconomic considerations in the health of urban areas. J Urban Health. 1998;75(2):228-235. doi:10.1007/BF02345090 9684234 PMC3456230

[30] Davis S, Malarcher A, Thorne S, Maurice E, Trosclair A, Mowery P; Centers for Disease Control and Prevention (CDC) State-specific prevalence and trends in adult cigarette smoking: United States, 1998-2007. MMWR Morb Mortal Wkly Rep. 2009;58(9):221-226. https://www.cdc.gov/mmwr/preview/mmwrhtml/mm5809a1.htm. Accessed January 27, 2020. 19282813

[31] Mokdad AH, Bowman BA, Ford ES, Vinicor F, Marks JS, Koplan JP The continuing epidemics of obesity and diabetes in the United States. JAMA. 2001;286(10):1195-1200. doi:10.1001/jama.286.10.1195 11559264

[32] Gebreab SY, Davis SK, Symanzik J, Mensah GA, Gibbons GH, Diez-Roux AV Geographic variations in cardiovascular health in the United States: contributions of state- and individual-level factors. J Am Heart Assoc. 2015;4(6):e001673. doi:10.1161/JAHA.114.001673 26019131 PMC4599527

[33] Christine PJ, Auchincloss AH, Bertoni AG, Longitudinal associations between neighborhood physical and social environments and incident type 2 diabetes mellitus: the Multi-Ethnic Study of Atherosclerosis (MESA). JAMA Intern Med. 2015;175(8):1311-1320. doi:10.1001/jamainternmed.2015.2691 26121402 PMC4799846

[34] Sussman JB, Halasyamani LK, Davis MM Hospitals during recession and recovery: vulnerable institutions and quality at risk. J Hosp Med. 2010;5(5):302-305. doi:10.1002/jhm.654 20533580

[35] Phelan JC, Link BG Controlling disease and creating disparities: a fundamental cause perspective. J Gerontol B Psychol Sci Soc Sci. 2005;60(spec No. 2):27-33. doi:10.1093/geronb/60.Special_Issue_2.S27 16251587

[36] McLaughlin DK, Stokes CS Income inequality and mortality in US counties: does minority racial concentration matter? Am J Public Health. 2002;92(1):99-104. doi:10.2105/AJPH.92.1.99 11772770 PMC1447397

[37] Kaiser Family Foundation Status of state Medicaid expansion decisions: interactive map. https://www.kff.org/medicaid/issue-brief/status-of-state-medicaid-expansion-decisions-interactive-map/. Published 2019. Accessed December 12, 2019.

[38] Clark CR, Ommerborn MJ, A Coull B, Pham Q, Haas JS Income inequities and Medicaid expansion are related to racial and ethnic disparities in delayed or forgone care due to cost. Med Care. 2016;54(6):555-561. doi:10.1097/MLR.0000000000000525 26974677 PMC5830941

[39] Cooper RA, Cooper MA, McGinley EL, Fan X, Rosenthal JT Poverty, wealth, and health care utilization: a geographic assessment. J Urban Health. 2012;89(5):828-847. doi:10.1007/s11524-012-9689-3 22566148 PMC3462827

