# Supplementary material for: Factors Associated With County-Level Variation in Premature Mortality Due to Noncommunicable Chronic Disease in the United States, 1999-2017
Source: JAMA Netw Open. 2020 Feb 28;3(2):e200241. doi: 10.1001/jamanetworkopen.2020.0241 (PMC7049090; doi:10.1001/jamanetworkopen.2020.0241)
Supplement: Supplement. — eFigure. Trends in County-Level, Age-Adjusted NCD Premature Mortality by Race/Ethnicity Groups, 1999-2017 eTable 1. Results of ITS on Trends in County-Level, Age-Adjusted NCD Premature Mortality by Income Groups in 4 US Regions, 1999-2017 eTable 2. Results of ITS on Trends in County-Level, Age-Adjusted NCD Premature Mortality by Race/Ethnic Groups, 1999-2017 [file jamanetwopen-3-e200241-s001.pdf]

Supplementary Online Content

Song S, Trisolini MG, LaBresh KA, Smith Jr SC, Jin Y, Zheng Z-J. Factors associated with county-level variation in premature mortality due to noncommunicable chronic disease in the United States, 1999-2017. 2020;3(2):e200241. *JAMA Netw Open*. doi:10.1001/jamanetworkopen.2020.0241

eFigure. Trends in County-Level, Age-Adjusted NCD Premature Mortality by Race/Ethnicity Groups, 1999-2017
eTable 1. Results of ITS on Trends in County-Level, Age-Adjusted NCD Premature Mortality by Income Groups in Four US Regions, 1999-2017
eTable 2. Results of ITS on Trends in County-Level, Age-Adjusted NCD Premature Mortality by Race/Ethnic Groups, 1999-2017

This supplementary material has been provided by the authors to give readers additional information about their work.

© 2020 Song S et al. *JAMA Network Open.*

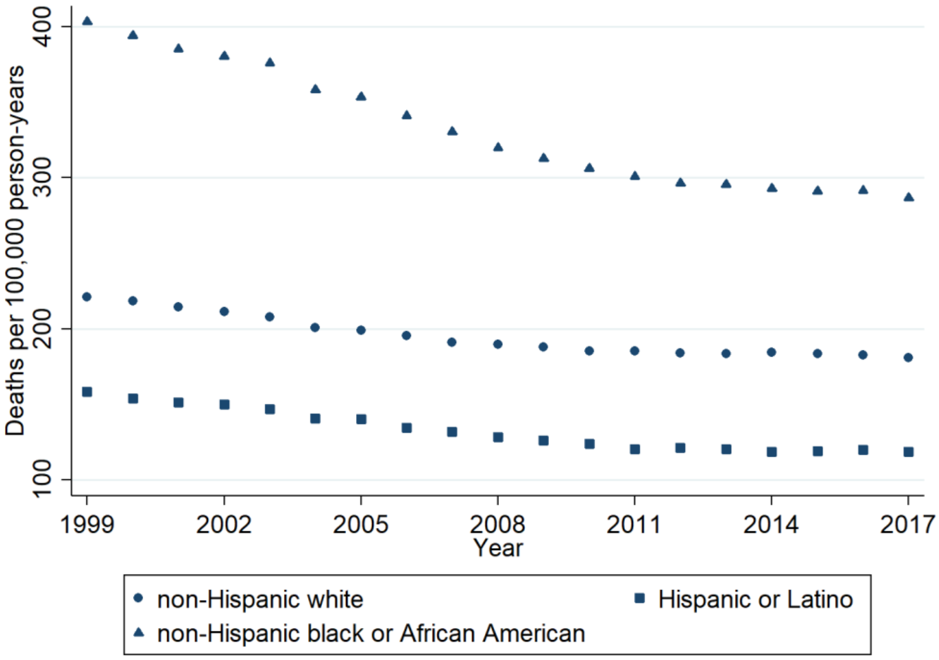

Note: Interrupted Time Series was used to test the trend of the mortality rate in each race/ethnic group. The mortality showed a significantly decrease trend in every race/ethnicity group from 1999 to 2010. The mortality showed a significantly slowly decrease trend in each group from 2010 to 2017. The results of ITS were showed in eTable 2.

eFigure. Trends in county-level, age-adjusted NCD premature mortality by race/ethnicity groups, 1999-2017

© 2020 Song S et al. *JAMA Network Open.*

eTable 1 Results of ITS on trends in county-level, age-adjusted NCD premature mortality by income groups in four US Regions, 1999-2017

Group	Trend prior 2010		Trend post 2010	
	Coefficient	p-value	Coefficient	p-value
Midwest & Low income	-1.84**	0.02	1.16	0.11
Midwest & Average income	-4.45***	<0.001	0.43**	0.04
Midwest & High income	-3.81***	<0.001	-0.22	0.54
Northeast & Low income	-3.25***	0.001	-0.24	0.53
Northeast & Average income	-3.06***	<0.001	0.73**	0.03
Northeast & High income	-3.65***	<0.001	-1.4***	<0.001
South & Low income	-3.37***	<0.001	3.51***	<0.001
South & Average income	-3.59***	<0.001	1.81***	<0.001
South & High income	-4.91***	<0.001	0.03	0.94
West & Low income	-2.88***	<0.001	1.70**	0.04
West & Average income	-3.61***	<0.001	0.38	0.44
West & High income	-2.38***	<0.001	-0.53	0.28

**p<0.05; ***p<0.01.

© 2020 Song S et al. *JAMA Network Open.*

eTable 2 Results of ITS on trends in county-level, age-adjusted NCD premature mortality by race/ethnic groups, 1999-2017

Group	Trend prior 2010		Trend post 2010	
	Coefficient	p-value	Coefficient	p-value
non-Hispanic white	-3.57***	<0.001	-0.53***	<0.001
non-Hispanic black or African American	-9.22***	<0.001	-2.41***	<0.001
Hispanic or Latino	-3.28***	<0.001	-0.61**	0.01

**p<0.05; ***p<0.01.

© 2020 Song S et al. *JAMA Network Open.*
